# Prediction of Strength Properties of Filling Packets in Selected Cooling Towers

**DOI:** 10.3390/polym13213840

**Published:** 2021-11-06

**Authors:** Monika Chomiak, Maciej Rojek, Józef Stabik, Małgorzata Szymiczek

**Affiliations:** Department of Theoretical and Applied Mechanics, Silesian University of Technology, Konarskiego 18A, 44-100 Gliwice, Poland; maciej.rojek@polsl.pl (M.R.); jozef.stabik@polsl.pl (J.S.); malgorzata.szymiczek@polsl.pl (M.S.)

**Keywords:** polypropylene films, degradation of polymeric materials, mechanical properties, infrared absorption spectrum, thermogravimetric curves, prediction of exploitation time

## Abstract

The operating conditions of thermoplastic polymer materials determine the changes in their functional properties. Accelerated aging tests do not give a full picture of the changes taking place in the polymer material, hence the conclusions drawn on the basis of exposure of these materials to damaging effects in real operating conditions are particularly important. The aim of the study was to determine the degree of degradation of polypropylene films used in the drainage blocks of cooling towers in a selected power plant in the Silesian voivodship, which allowed forecasting the operating time over a period of 10 years. A number of 600 mm high drip blocks were tested, on which 300 mm high blocks were mounted. The tests were carried out on films subjected to the aging process in the conditions of continuous operation of a cooling tower (almost 100% humidity). The water flow is accompanied by heat exchange, the side effect of which is deposits formation on the surface of the drip blocks, negatively affecting the operation of the cooling tower. The degree of degradation resulting from operational aging was assessed on the basis of the strength properties determined in the static tensile test, thermogravimetric analysis and FTIR spectra. Changes in properties during operation were determined on the basis of the obtained results of the strength tests, which were compared with the tensile strength and elongation at break of reference samples (not subjected to aging in the operating conditions of cooling tower drip blocks). The obtained results were related to the properties of the reference samples not subjected to the degradation process. Based on the collected data, the tensile strength and deformation at fracture after a 10-year service life were predicted.

## 1. Introduction

**Figure 1:**
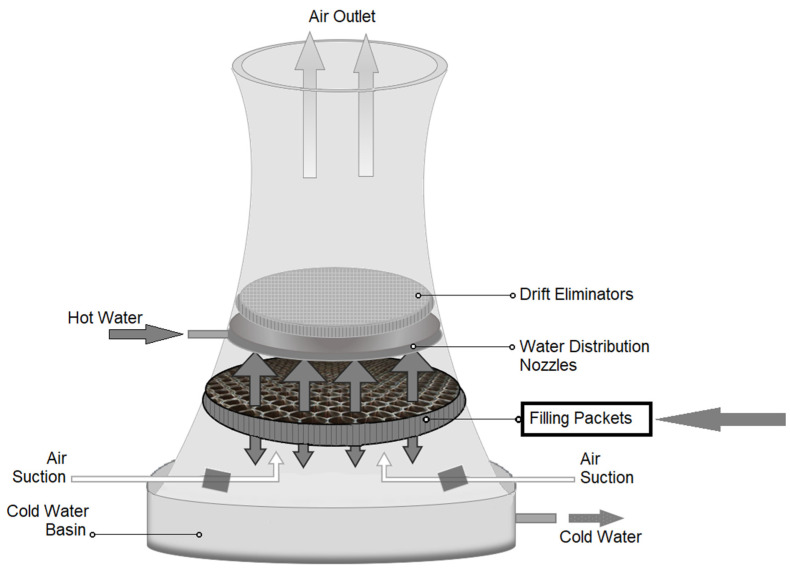
Schematic view of the natural draft cooling tower.

The basic problem of the polymeric materials application, often operating in very heavy environmental conditions, is determining their durability [1,2,3]. It is a feature conditioned by the aging of the material, which occurs gradually and most often leads to irreversible changes in the material; this has a significant impact on the performance characteristics directly related to reliable and safe operation. Determining the degree of degradation of polymeric materials is a difficult and time-consuming task. Conducting tests in the accelerated aging mode, in accordance with the time–temperature equivalence principle, only allows for an estimate of the durability. Therefore, the results of research conducted under real conditions are particularly important [4,5,6,7]. In the case of cooling towers, the degradation factors are the effects of heat, solar radiation (UV), cooled water and microorganisms [8]. Figure 1 shows a typical cooling tower structure. A cooling tower is an open direct-contact-type heat exchanger where hot water from a system or condenser gets cooled by direct contact with fresh air. Cooling towers use the principle of evaporation of water against the air flow. Hot water is sprayed from the nozzles for increasing the heat transfer surface area. The temperature and humidity of the air get increased after direct contact heat transfer between hot water and fresh air. The warm and moist air being less dense goes to the top of the tower, and cold water gets collected at the bottom of the tower. Fresh air is supplied from the bottom of the cooling tower due to the density difference between hot air inside the stack and atmospheric air outside the cooling tower. Such an environment is conductive to the formation of sediments and the development of microorganisms that reduce the cross-section of the channels of the fill materials (i.e., drip blocks), which significantly affects the efficiency of work and operating conditions. The performance of a cooling tower is determined by many factors, among which the cooling tower fill is of great importance. The fills types and quality decide whether a cooling tower is able to transfer heat efficiently or not. By choosing the right type of cooling tower fill, water will flow in much more surface area and the hot water will be cooled quickly. Cooling tower fill is made of PVC or PP plain plate by specialized machine [9,10,11,12,13,14,15,16,17,18].

A number of works on accelerated aging of polypropylene and lifetime prediction can be found in the literature. For example, after that reviewing independent data for the thermal degradation of PP, Celina [19] showed that a transition temperature of ~83 °C existed, with the high temperature process having a considerable higher activation energy (107–156 kJ/mol) than the low temperature process (35–50 kJ/mol). Such examples demonstrate the excellent fits that can be obtained by introducing at a minimum two processes to address the curvature in Arrhenius plots. For accelerated aging studies where evidence of some curvature exists but limited data are available, better lifetime predictions could be made by estimating a low temperature process activation energy or allowing for a second rate dependence instead of forcing a straight line extrapolation. Research conducted by Gahleitner et al. [20] showed that the aging process of PP is determined by the nature of the polymer, the type and conditions of conversion and the temperature level of aging. The cumulative effects of the crystallization behavior of the polymer and cooling history determine the crystallinity and superstructure of the formed article, which in turn determines the aging behavior. On the temperature scale, two or (for an impact copolymer) three clear transitions related to the mobilization of parts of the material can be identified, which clearly limits the applicability of accelerated tests at elevated temperatures to predict long-term behavior. Similar conclusions were drawn in the study in [21], which investigated the effect of aging at elevated temperature on microstructural changes in the isotactic polypropylene matrix. The degree of crystallinity was found to increase after aging at both 90 and 140 °C. In turn, in [22], studies of a random polypropylene copolymer with the addition of ethylene or butylene conomer with differentiated structure α-monoclinic, β-hexagonal) were carried out. Aging was carried out at the temperatures of 95 and 135 °C for 750 days. The strain at break decreased during the first 500 h and was attributed to the physical mechanisms of aging. Better aging behavior was demonstrated by PP with the addition of ethylene of the β structure. It follows that the aging behavior of polypropylene is primarily influenced by the structure of the material and the degree of crystallinity. Fiebig [3] found changes in the amorphous and/or mesomorphic regions of the material at room temperature, which led to an increase in the density and modulus. In the case of elevated temperatures, a relaxation and a recrystallization take place, which have a positive effect on the temperature of deflection and the impact toughness.

The presence of a side group in the polymer chain determines the thermo-oxidation susceptibility of polypropylene. The process of this degradation is a typical free-radical chain cutting process. In this process, there are four stages: initiation, cutting propagation, branching and termination with the formation of nonradical products. A great many different reactions are proposed in the chemical description of the individual steps. There are also many methods of stabilizing and preventing the thermo-oxidation processes of polypropylene [23,24]. The mechanisms of degradation of thermoplastic polymer materials have been described, inter alia, in [25,26,27,28,29,30,31,32,33,34]. However, it is difficult to find in the literature, studies on the natural aging of polypropylene working in cooling towers, where there is a specific operating environment with 100% humidity and a variable temperature.

The aim of the study was to determine the effect of long-term operation in the cooling towers of a selected power plant from Upper Silesia on the functional properties of polypropylene films used in cooling tower drip blocks. For this purpose, observations were made of changes in selected properties of materials under the influence of the atmosphere of cooling towers and chilled water: air with a temperature higher than the ambient temperature, full humidity, microorganisms, water pollution and chemicals used for its disposal, and sediments formed on their surface. To study the impact of specific operating conditions and, at the same time, aging conditions, i.e., heat and humid environment, on the structure and properties of polypropylene, the method of infrared spectrophotometry (FTIR) as well as thermogravimetry (TGA) and strength tests were used. The research carried on these mechanical properties was used to forecast changes in the properties of polypropylene products during the assumed 10-year service life. The aging studies carried out in the work environment are particularly important for the design of drip blocks and forecasting the lifetime of these elements and changes in operational properties in a given time-period. Natural aging tests minimize the error resulting from the lack of synergistic interaction of many degradation factors, which are difficult to simulate in accelerated aging. Hence, these arguments prove the originality of the research topic undertaken.

## 2. Experimental

### 2.1. Preparation of Samples for Tensile Strength Tests

**Figure 2:**
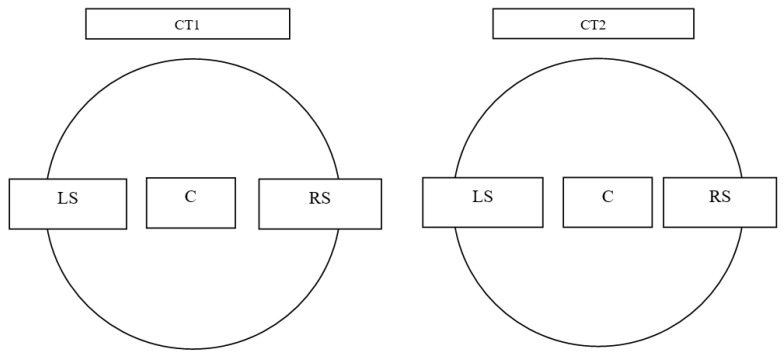
Block location diagram.

The test samples were obtained from drip blocks of two cooling towers (marked CT1 and CT2) from the Silesian voivodeship, which are characterized by specific climatic conditions. Test samples (filling packets with drip blocks) with a height of 600 mm (A) and 300 mm (B) were taken from different areas, as shown in Figure 2. The samples were marked as shown in Figure 2, where LS refers to the left side, C the center and RS the right side.

**Figure 3:**
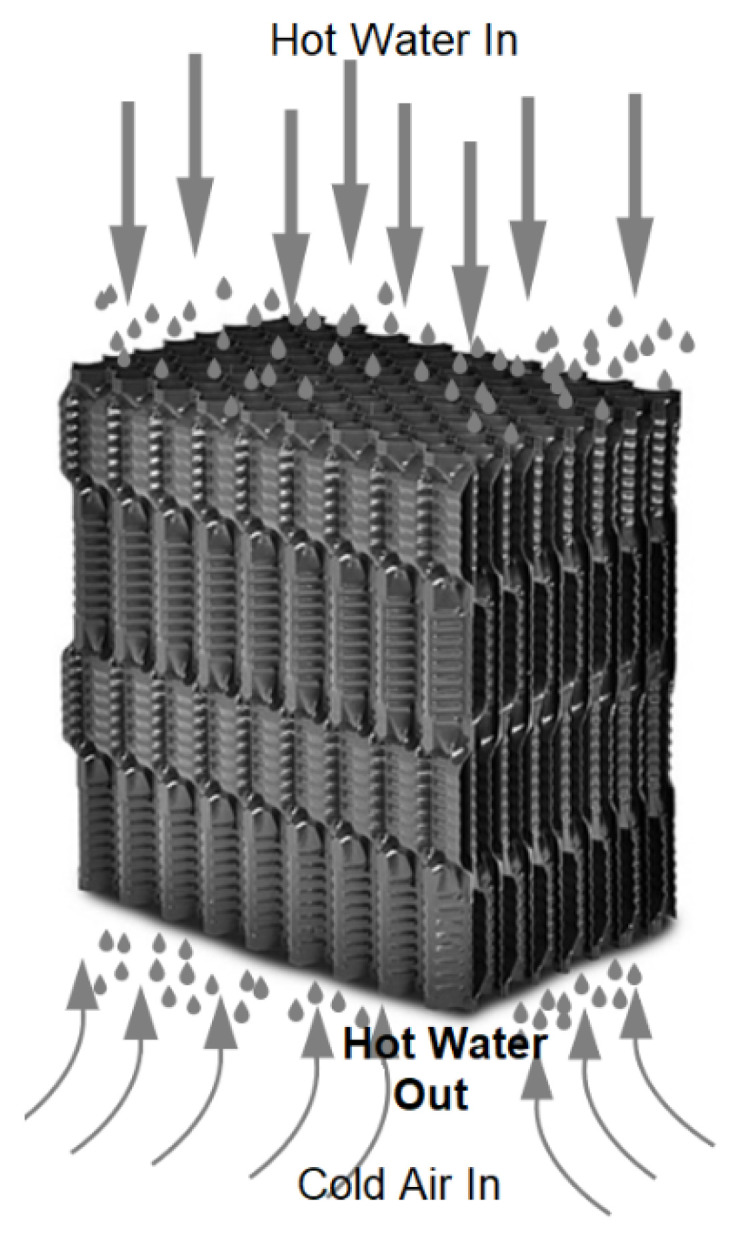
Working principles of cooling tower fills.

In the test object, a block with a height of 600 mm was installed in the lower area, and a block with a height of 300 mm was placed on it, which, in total, resulted in a 900 mm high filling. Aging was carried out in two cooling towers with fills with straight channels through which water flows, giving up the heat (Figure 3).

The heat transfer process is accompanied by the precipitation of sediments on the surface of the sprinkler. In the tested cooling towers (with natural draft), an increase in sediment and corrosion was observed compared to other facilities of this type outside of Silesia. This is related to the parameters of the circulating water, which is the cause of deposits and corrosion. The tested blocks were mounted in a cooling tower, in which there were two work zones, internal and external. The operating conditions of the blocks throughout the cooling tower were identical.

Reference samples were taken at the stage of installing the drip blocks and stored in conditions that prevented the effects of UV radiation, chilled water, microorganisms and work at elevated temperature.

The assumed service life of polypropylene in the conditions in which the reference samples were stored is at least 50 years [35], which is mainly the result of the lack of UV radiation (polypropylene is sensitive to radiation with a wavelength of 370 nm) [36,37]. Providing appropriate storage conditions allowed the assumption that the properties of the reference samples would not change significantly over the 7-year storage period. The value of the deformation at break of random polypropylene, not subjected to storage and aging processes, is approximately 483%. This value differs from that declared by the manufacturer BASSEL, and this is due to the drip block shaping process, in which the material is first extruded, vacuum-formed, ultrasonically welded and finally heat-treated for straightening. The elongation at break for the reference samples is 459.19%. Thus, the difference between the reference samples and the produced samples (not subjected to the storage process) amounts to approximately 5%. Thus, accepting the samples in storage as the reference samples generates some error, but this does not affect the lifetime forecast made in Section 3.3. At the same time, due to the operating conditions and the specific structure of the fill channels, the manufacturers guarantee their correct operation for 10 years. It is related to the formation of sediments on the canals that significantly limit the flow of water mist. It should be noted that cleaning the fill materials with surfactants would restore efficiency and extend the service life, but it is a complicated and time-consuming process. 

**Figure 4:**
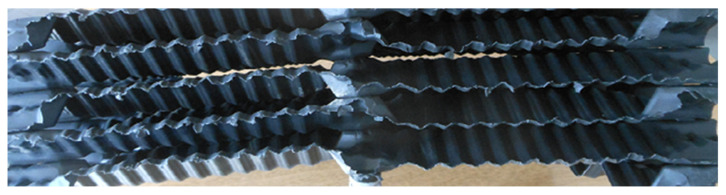
View of the filling packets of the cooling tower.

**Figure 5:**
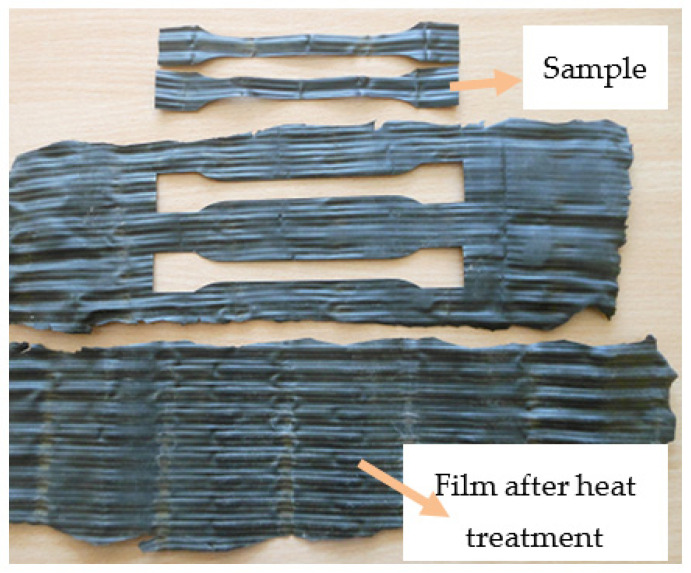
View of the straightened film sheets.

Before starting the research, the elements of the filling packets installed in the cooling towers were subjected to a cleaning process to remove sediments resulting from the influence of water and developing microorganisms. The complex geometry of the drip blocks (Figure 3 and Figure 4), obtained in the thermoforming process, made it impossible to cut out samples for mechanical tensile testing. Therefore, after separating the blocks into sheets, a heat treatment of the single sheets was carried out at a temperature of 160 °C for 15 min. The purpose of the heat treatment was to reverse the effects of deep thermoforming by using the phenomenon of elastic recovery (shape memory) at elevated temperature. The heat treatment conditions were selected experimentally, and as a result of this treatment, it was possible to almost completely revert to a flat film before thermoforming, but only immediately after the forming process. With the passage of time, the stresses applied to the film and “frozen” through rapid cooling are subject to the relaxation phenomenon. As a result, the film after heat treatment was a little wavy and was additionally pressed between two heated steel plates. Figure 5 shows the straightened film, prepared for cutting out the samples. The adopted method of sample preparation allowed us to minimize the effects of deep embossing.

### 2.2. Research Methodology

**Figure 6:**
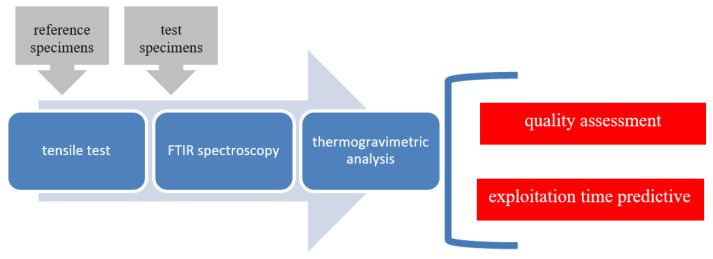
Research scheme.

In order to forecast selected strength properties for the 10-year service life of the drip blocks, as assumed by the designer of the cooling tower, tests were carried out to determine the strength characteristics in a tensile test, spectroscopic tests and a thermogravimetric analysis. The research scheme is presented in Figure 6. In the final stage, the properties were forecasted over a period of 10 years on the basis of tensile strength and deformation to fracture.

#### 2.2.1. Strength Tests

**Figure 7:**
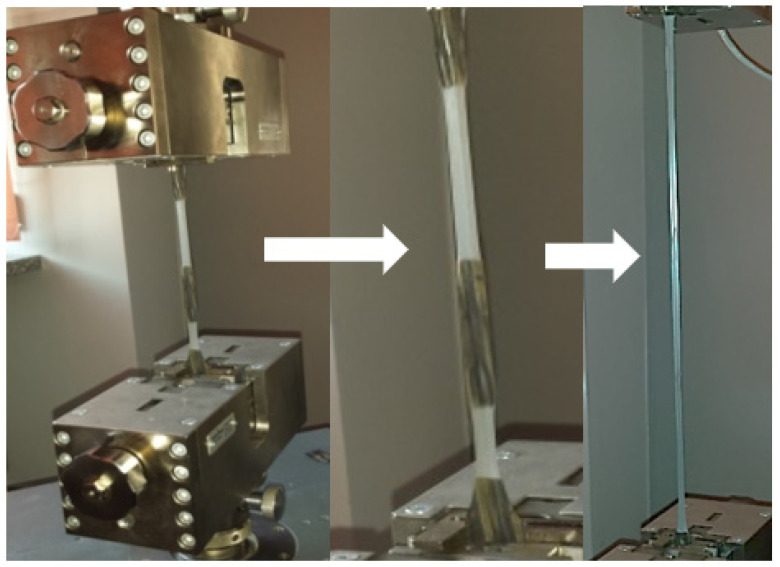
View of the sample during tensile test.

A static tensile test was carried out using the Zwick/Roell Z020 (Zwick GmbH & Co. KG, Ulm, Germany) testing machine, in accordance with the PN-EN ISO 527 standard [38]. The test specimens were prepared by punching in accordance with PN-EN ISO 3167: 2005, type A [39]. The tensile speed was 50 mm/min. Based on the obtained results, the tensile strength and strain at break were determined. During the tests, unevenly appearing neckings were observed (Figure 7), which are related, among others, to the process of thermoforming and sample preparation. As can be observed, the process of sample preparation had a significant influence on the formation of a neck. In the first stage, the narrowing was observed for the areas with the greatest embossment.

#### 2.2.2. Spectroscopic Test

Spectroscopic tests were carried out using the reflection method by means an infrared spectrometer by Thermo Scientific, type Nicolet 6700 (Thermo Fisher Scientific, Waltham, MA, USA), using the ATR attachment. The test samples were prepared using the punching method from the fillings of the aged and reference filling packets. Samples for spectroscopic examinations were taken from elements that had not been heat treated but had been cleaned of sediments and microorganisms.

#### 2.2.3. Thermogravimetric Research

Thermogravimetric tests were carried out by means of a Mettler Toledo TGA (Switzerland, Greifensee) apparatus. Thermogravimetric analysis consisted in measuring the weight of the sample while heating it at a constant rate of 20 °C/min. The obtained diagram of the dependence of the weight of the sample on the temperature allowed to determine the temperatures of transitions taking place during the thermal decomposition of the tested material. Samples for thermogravimetric tests were cut from blocks that were not subjected to heat treatment.

## 3. Results and Discussion

### 3.1. Analysis of the Results of Endurance Tests

**Figure 8:**
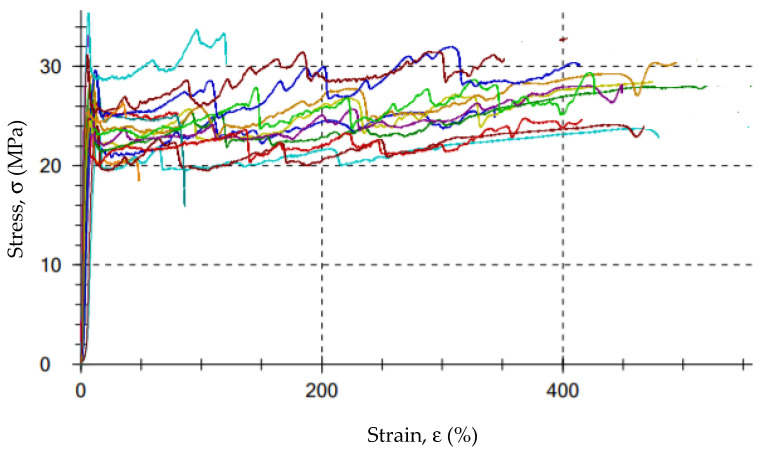
An example of the stress–strain diagram for a selected series of samples.

Figure 8 shows the stress–strain curves for a selected series of samples. As can be seen, all the curves have a characteristic “serrated” course resulting from the geometry (wavy surface resulting from thermoforming, Figure 5) and the sample preparation process. Due to the relatively large observed scatter of the results of the examined characteristics, at least 10 measurements were carried out for each series in order to average the results for further analyzes. It allowed us to minimize the spread to approximately 20% for the tensile strength and approximately 32% for the elongation at break.

**Figure 9:**
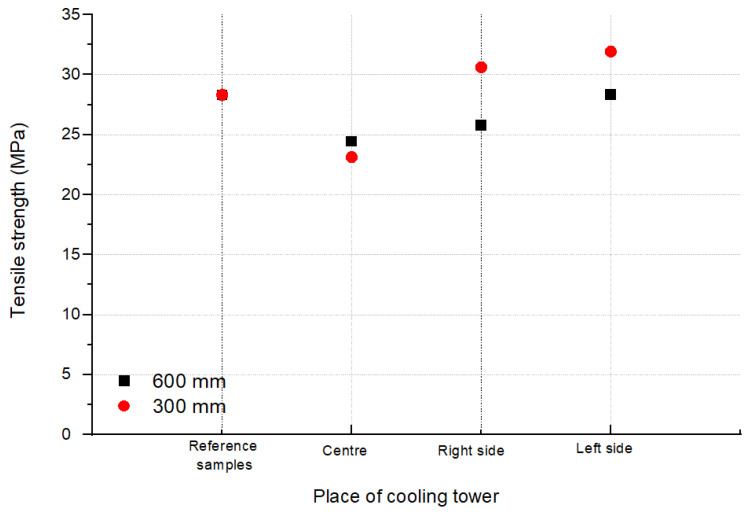
Tensile strength from block positions according to Figure 2 for CT1.

**Figure 10:**
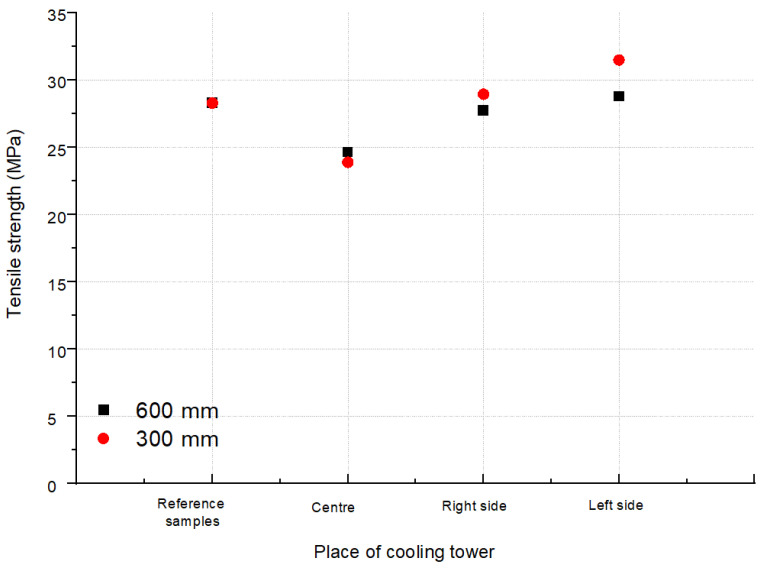
Tensile strength from block positions according to Figure 2 for CT2.

**Figure 11:**
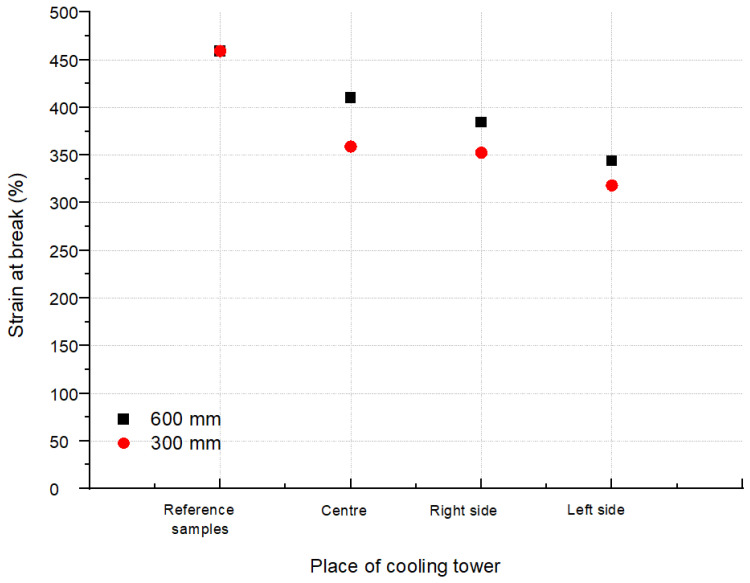
Strain at break from blocks positioned as shown in Figure 2 for CT1.

**Figure 12:**
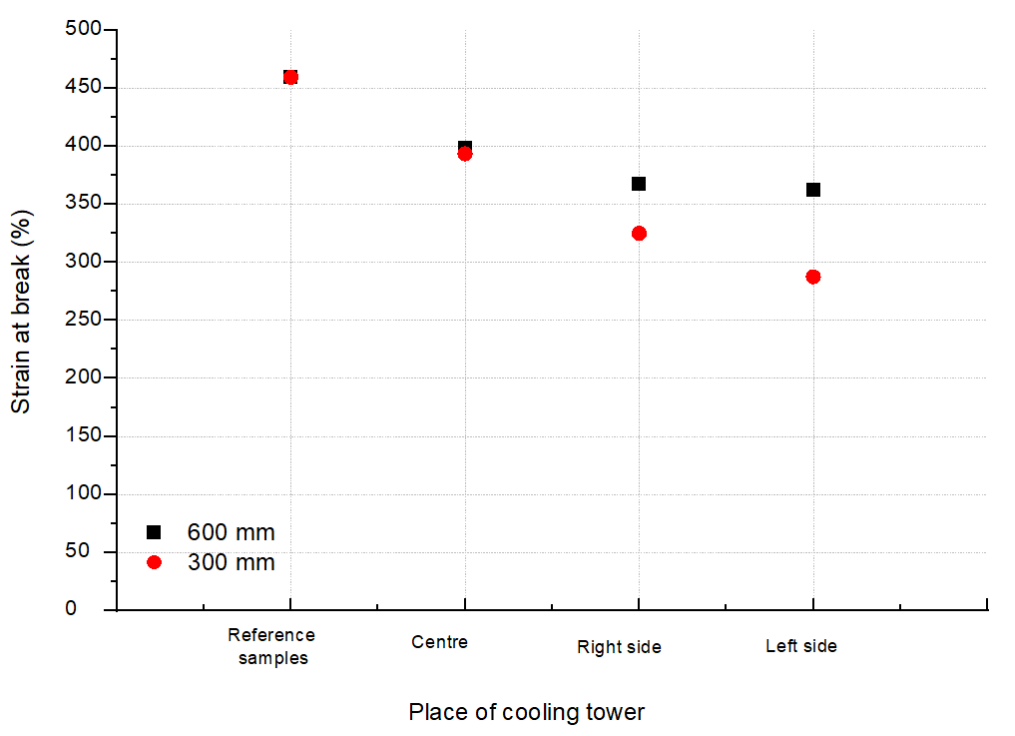
Strain at break from blocks positioned as shown in Figure 2 for CT2.

The averaged values of the stresses at break for the tested areas (L, R and C according to Figure 2) of the CT1 and CT2 cooling towers are shown in Figure 9 and Figure 10. Figure 11 and Figure 12 show the average values of the elongation at break for the tested drip blocks. The presented samples were compared to the reference samples (stored, obtained from the same batch of material).

When analyzing the obtained results, no clear correlation was observed between the history of the samples and their location (as shown in Figure 2) and the tensile strength and strain at break. The observed results scatter arises in connection with the method of sample preparation, which is significantly influenced by the processes of heat treatment, film straightening, but most of all, by material degradation under operating conditions. The effect of degradation is, firstly, the cutting of the thermoplastic chains, which causes the chains to be less intertwined, leading to a reduction in the modulus of elasticity and strength. Secondly, the free radicals formed during the breaking of the chains have the ability to attach many different active substances from the environment or the composition of the material, which can stiffen the chain, increase its polarity and increase the interactions between the chains, and thus increase the value of the elastic modulus. Thirdly, degradation processes may lead to the detachment of the –CH3 side group. Chain fragments can be inserted between the active sites created in this way, and such chains can also join directly. In each case, cross-linking of the polymer occurs, resulting in an increase in the value of the modulus of elasticity. Depending on which one of the mechanisms is dominant, the value of the modulus of elasticity is increased or decreased. In most cases, as the degradation processes progress, the modulus of elasticity increases with a simultaneous increasingly brittle behavior of the material during fracture. Therefore, changes in the modulus of elasticity are most often not taken as a measure of the progress of the degradation process. Furthermore, in the described tests, the modulus of elasticity was not determined. 

The obtained tensile strength results and the fact that all the samples broke with large plastic deformations indicate that both the reference samples and the samples taken from the cooling towers have not yet reached the inflection point on the aging curve, characteristic of the aging of many polymers [40] and are suitable for further use.

The slight difference in strength between the reference samples and those operated in cooling tower indicates that the aging process is not yet advanced. Samples with a strength significantly lower than the others were subjected to a careful analysis and indicated significant shape errors, especially on the side edges. For thin film samples, any shape errors on the side surface are notches and errors covering the entire thickness of the sample. From such notches and defects, cracks develop at stress levels much lower than the actual strength of the material under test. 

The analysis of the strength results of the tested samples (tensile strength, tensile elongation), due to the too large dispersion of the results, did not allow for an unequivocal determination of the changes in the properties of the drip blocks operated in the cooling towers. It can be observed that two reference samples show worse strength properties in relation to the used samples, which is unlikely, but cannot be ruled out.

It should be noted that the samples taken from the central area show the lowest tensile strength values and at the same time the highest elongation at break after a 7-year service life. The differences between the reference sample and the sample taken from the central part are approximately 11% for 600 mm blocks and 23% for 600 mm CT1 blocks and 15% for CT2. For the samples LS and RS, regardless of the type of cooling towers, greater differences in strength are visible, with 16% for RS 600 mm CT1, 24% for RS 300 mm CT1, 20% for RS 600 mm CT2, and almost 30% for RS 300 mm CT2 in relation to the reference samples. Even greater changes are observed for the samples taken from the left side of the cooling tower. The differences are 25% for the LS 600 mm CT1, 30% for the LS 300 mm CT1, 25% for the LS 600 mm CT2, and 37% for the RS 300 mm CT2 less than the reference samples. Blocks with a height of 300 mm, regardless of the area, show a lower elongation at break values than blocks of 600 mm, regardless of the cooling tower in which they worked. The differences between the right and left side may be caused by external conditions or air circulation in the cooler itself. 

### 3.2. Analysis of the Results of Structure Research

**Figure 13:**
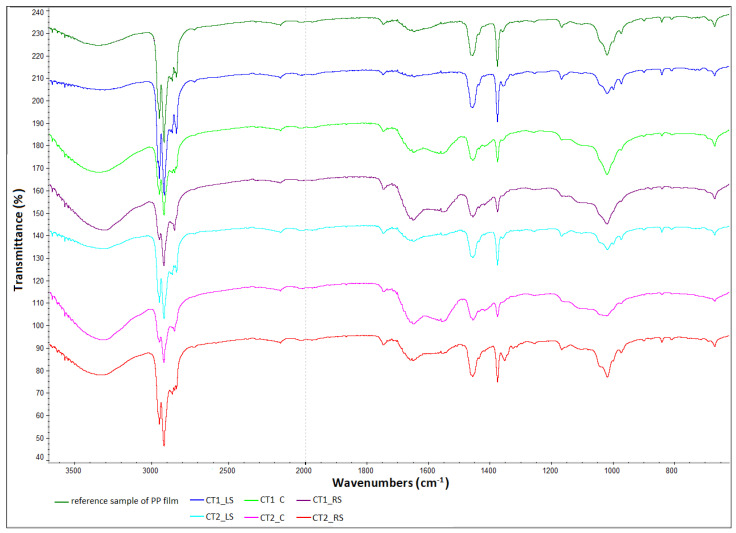
Summary of polypropylene spectra in relation to the reference spectrum.

Figure 13 compares the obtained FTIR spectra of polypropylene samples collected from various areas of exploitation with that of the reference sample. The remaining spectra are included in the Supplementary Materials (Figures S1–S5).

Changes in the intensities and shifts of some absorption bands can be noticed in the spectra of the samples exposed to thermal aging in relation to the reference samples.

In the spectra of aged samples one can see a decrease in the intensity of the bands at the wavenumbers 1380 cm^−1^ (bending vibrations δs of the CH_3_ group), 1153 cm^−1^ (stretching vibrations υC–C(CH_3_)) and 964 cm^−1^ (vibrations related to the presence of syndiotactic polar-zigzag conformation) compared to the intensity of these bands in the spectra of the control samples.

In addition, in the wavenumber range 2840–2880 cm^−1^ (υCH_2_-, υCH_3_- vibrations) changes can be seen in the composition of the absorption bands for aged samples compared to the control samples. The vibration range of the 1740 cm^−1^ carboxyl group shows similarities for all samples, which may indicate a slight oxidative degradation of the samples.

Thermal aging probably caused changes in the structure of the polymer chain—it cracks into smaller fragments.

The analyzed spectra indicate changes in the intensity of individual peaks of the absorption spectra in relation to the reference sample. The greatest changes are observed for the CT1_C sample, and much smaller changes for the CT1_LS and RS as well as CT2_C and RS samples, which confirms the changes observed in the strength characteristics

**Figure 14:**
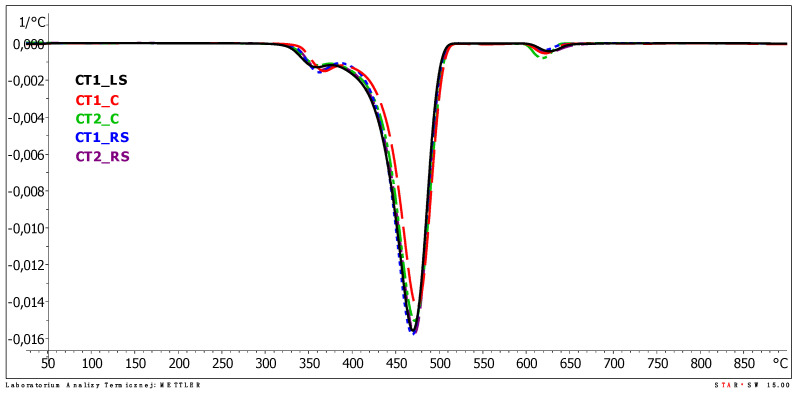
DTG curves of tested samples.

**Figure 15:**
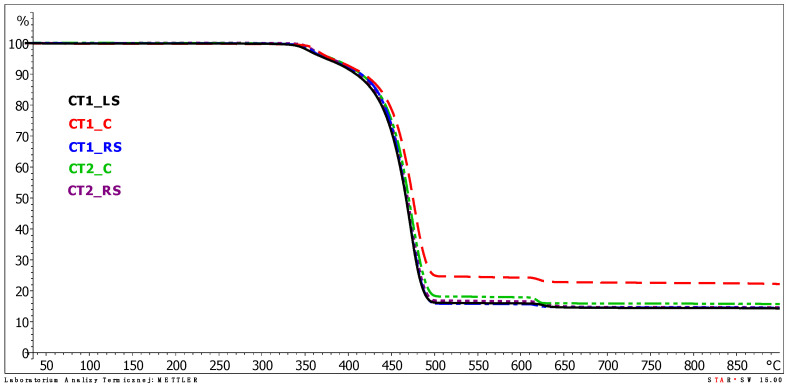
TGA curves of tested samples.

**Figure 16:**
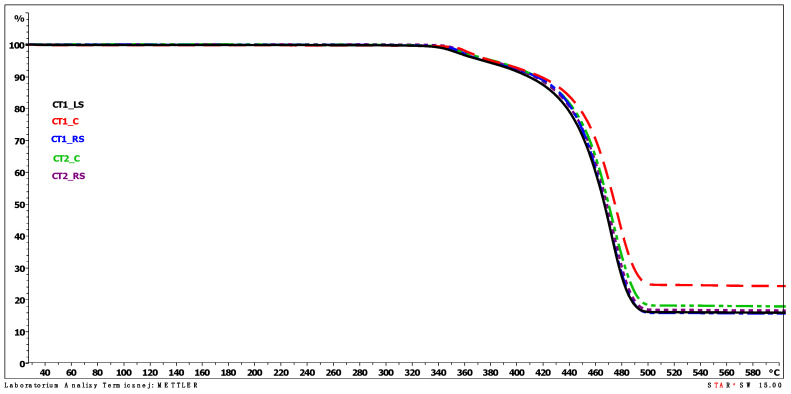
TGA curves of tested samples—temperature range from 25 to 600 °C.

The results of the thermogravimetric analysis (TGA) are presented graphically in the plots from Figure 14, Figure 15 and Figure 16. The individual TGA curves for the dependence of the sample mass on the temperature increasing with a constant speed of all the tested areas of the first and second cooling towers are presented in the Supplementary Materials (Figures S6–S10).

Table 1 presents the characteristic temperatures of changes occurring during the decomposition of the material while heating the samples at a constant rate of 20 °C/min. Table 2 presents the sample mass loss at particular mass loss steps and the sample mass residue at the final temperature of the TGA analysis.

The results of the thermogravimetric analysis are consistent for samples taken from different areas. The analysis of the TGA curves indicates that polypropylene films undergo decomposition with a two-step mass loss in an inert gas atmosphere. The first mass loss was determined in the temperature range of about 280–380 °C. It was about a 5% (m/m) sample mass loss for all the samples tested, with the lowest loss of 4.2% for sample CT2_C and the highest loss of 5.9% for sample CT1_RS. The peak value for the first mass loss (the temperature at which the mass loss occurred at the highest rate, T_max1_) was determined on the DTG curve. For all tested samples, the values of T_max1_ were very close to each other and occurred in the temperature range from 358.7 to 364.3 °C. The mentioned values were determined for samples CT1_LS and CT1_C, respectively.

In the second weight loss recorded on the TGA curves, which is a much higher sample mass loss than the first one, polypropylene decomposition occurs. The temperature determined as the peak maximum value on the DTG curve (T_max2_) for the second weight loss ranged from 466.8 to 471.4 °C for the samples CT1_RS and CT1_C, respectively, which are very similar values. The lack of significant differences in T_max2_ values leads to the conclusion that the location of the film in the cold storage does not significantly affect the acceleration of samples degradation (thermo-oxidative degradation).

The temperature at sample mass losses of 3%, 5%, 10% and 15% of the initial mass of the sample did not differ significantly between the samples tested, except for sample CT1_C for which the temperature values determined at 3% and 5% mass loss were about 10 °C higher than the sample with the lowest temperature values (CT1_LS). A summary of the temperature values at the specified sample weight loss is shown in Table 2. During TGA analysis in an oxygen atmosphere (temperature range of analysis at this stage was from 600 to 900 °C), a third weight loss was observed in the temperature range from 600 to approximately 640 °C. The third mass loss in an oxidizing gas atmosphere is related to the decomposition of carbon black, which may have been added to the polypropylene plastic during the film manufacture, e.g., used as a coloring agent of the plastic, as well as that generated during the decomposition of the polymeric material that took place in an inert gas atmosphere. The temperature value determined at the peak maximum on the DTG curve related to the third weight loss (T_max3_) was in a narrow range from 621.8 to 627.9 °C. The value of the third mass loss ranged from 1.2 to 2.2% (m/m).

The residue after decomposition determined at 900 °C was about 14.3–15.7% m/m, except for sample CT1_C for which the value of residue after decomposition was higher and was 22.8%. The higher value of residue after decomposition for one sample, located in the central part of the cold store, may be related to a higher content of contaminants of inorganic origin that may have settled on both sides of the film during its exploitation. This phenomenon may be favored by the elevated temperature in the cooling tower, which makes polypropylene more flexible and susceptible to microdamage by inorganic contaminants being driven into the film surface than at 20 °C.

The evaluation of the results of TGA analyses shows that irrespective of the location of the film in the drip blocks of the chimney linings, the value of the decomposition temperature remains similar. No significant effect of the polypropylene film location on the course of its decomposition is observed, and more importantly no significant decrease in the value of the decomposition temperature (T_max2_) is observed for any of the tested samples. All the tested samples show the same amount of sample mass loss. The location of the film in the drip blocks of the cooling tower, especially in its central part, slightly affects the values of the temperature and the mass loss characterizing only the first mass loss, and the sample remaining after decomposition of the sample coming from only one of the two analyzed cooling towers. The slight differences noted may be related to the operating conditions of the cooling tower from which sample CT1_C was taken.

The obtained results do not show any significant differences between the individual measurements.

### 3.3. Forecast of Durability and Suitability for Further Operation of the Drip Blocks

**Figure 17:**
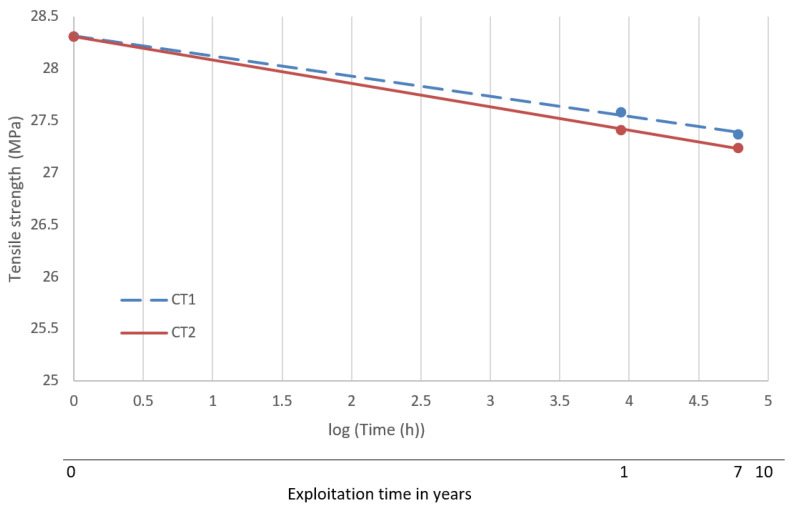
Relationship between tensile strength and operation time for cooling towers 1 and 2.

**Figure 18:**
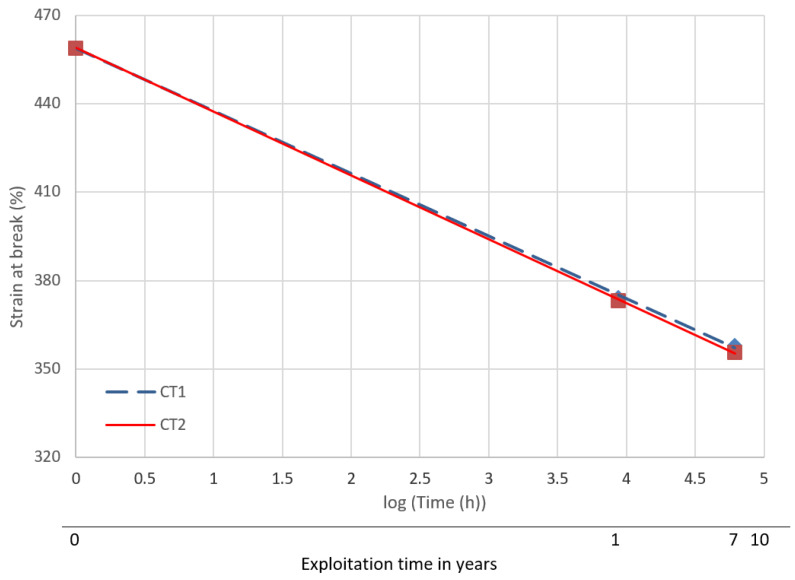
Strain at break dependence on operation time for cooling tower 1 and 2.

Forecasting was performed using statistical methods referring to the averaged results of mechanical tests of the reference samples and after one year and seven years of operation. The results were approximated by the exponential function [41]. Figure 17 shows the approximation functions of the tensile strength values against the aging time. Figure 18 shows the approximation functions of the strain at break. The lifetime of the drip blocks was assumed to be 10 years.

For the cooling tower 1, the approximation function takes the form:

Tensile strength [MPa] = −0.193log(Time[h]) + 28.315, R^2^ = 0.99 (1)

and for cooling tower 2:

Tensile strength [MPa] = −0.225log(Time[h]) + 28.308, R^2^ = 0.99 (2)

For the cooling tower 1, the approximation function takes the form:

Strain at break [%] = −21.237log(Time[h]) + 458.82, R^2^ = 0.99 (3)

and for cooling tower 2:

Strain at break [%] = −21.675log(Time[h]) + 458.93, R^2^ = 0.99 (4)

Based on the determined approximation functions, the expected values of the tensile strength and deformation at fracture after 10 years of operation of the cooling towers were determined. The obtained results are summarized in Table 3.

## 4. Conclusions

Based on the conducted research, it can be concluded that:

- The strength properties depend on the area of operation. The test samples taken from the central part were characterized by the lowest tensile strength and the highest elongation. It may be the result of an uneven impact of the exploitation environment. The outer areas showed the higher strength, with the left side of the cooler showing the highest values. The spectra obtained confirmed the greatest changes were in the CT1_C region (a decrease of intensity in relation to the spectrum of reference samples). This is related to the effect of heat (increased temperature) in the central part of the drip blocks, and this additionally favored the formation of sediments.
- The determined dependency functions (Formulas (1)–(4)) make it possible to forecast the strength properties in the assumed 10-year period of operation in cooling towers. It was observed that the change in strain at break was about 23%, while for the tensile strength it was about 4%. The size of the changes was also confirmed by the intensity of the FTIR spectra in characteristic areas (i.e., 964, 1153, 1380 and 2840–2880 cm^−1^) and by thermogravimetric analysis The observed changes were linear in the adopted coordinates and the approximation curves did not show a break inflexion, which forecasts a safe operation of the drip blocks in the assumed period of 10 years.
- The evaluation of the results of the TGA analyses shows that irrespective of the location of the film in the drip blocks of chimney linings, the value of the decomposition temperature remains similar. No significant effect of the polypropylene film location on the course of its decomposition was observed, and more importantly no significant decrease in the value of the decomposition temperature (Tmax2) was observed for any of the tested samples.
- The location of the film in the drip blocks of the cooling tower, especially in its central part, slightly affects the values of the temperature and mass loss characterizing the first mass loss only and the sample remaining after decomposition of the sample coming from only one of the two analyzed cooling towers.

## Figures and Tables

**Table 1:** Sample mass loss step temperatures read from the DTG curves from thermogravimetric analysis and temperatures of specified sample mass loss.

Sample Determination	Temp. of the First Mass Loss Step (°C)	Temp. of the Second Mass Loss Step (°C)	Temp. of the Third Mass Loss Step (°C)	Temp. at 3% (M/M) Sample Mass Loss (°C)	Temp. at 5% (M/M) Sample Mass Loss (°C)	Temp. at 10% (M/M) Sample Mass Loss (°C)	Temp. at 15% (M/M) Sample Mass Loss (°C)
	T_max1_	T_max2_	T_max3_	T_3%_	T_5%_	T_10%_	T_15%_
CT1_LS	358.7	468.1	627.9	357.6	373.7	408.3	426.8
CT1_C	367.6	472.8	623.8	369.1	386.0	419.5	435.9
CT1_RS	361.5	466.8	621.8	360.3	373.9	414.0	431.3
CT2_C	360.7	469.8	623.8	362.4	380.9	414.0	431.5
CT2_RS	362.3	470.0	627.2	363.7	379.2	412.7	429.9

T_max_—measured temperature from DTG.

**Table 2:** Sample mass loss determined during TGA and sample mass residue at 900 °C.

Sample Determination	First Mass Loss (%) (m/m)	Second Mass Loss (%) (m/m)	Third Mass Loss (%) (m/m)	Mass Residue at 900 °C
	m_L,1_	m_L,2_	m_L,3_	m_R,900_
CT1_LS	5.5	78.6	1.6	14.3
CT1_C	5.4	70.0	1.8	22.8
CT1_RS	6.2	78.2	1.2	14.4
CT2_C	4.4	77.7	2.2	15.7
CT2_RS	5.6	77.8	2.0	14.6

**Table 3:** Predicted strength properties.

Property	CT1	CT2
Tensile strength (MPa)	27.36	27.19
Strain at break (%)	353.91	351.86

## Data Availability

Not applicable.

## Supplementary Materials

The following are available online at https://www.mdpi.com/article/10.3390/polym13213840/s1, Figure S1: The spectrum of CT1_L; Figure S2: The spectrum of CT1_C; Figure S3: CT1_RS spectrum; Figure S4: The spectrum of CT2_C2; Figure S5: CT2_RS spectrum; Figure S6: Dependence of sample mass on temperature increasing with constant velocity in the TGA CT1_LS tests; Figure S7: Dependence of sample mass on temperature increasing with constant velocity in the TGA CT1_C tests; Figure S8: Dependence of the sample mass on the temperature increasing with a constant speed in the TGA CT1_RS tests; Figure S9: Dependence of sample mass on temperature increasing with constant velocity in TGA CT2_C tests; Figure S10: Dependence of sample mass on temperature increasing with constant velocity in TGA CT2_RS tests.
