# Supplementary material for: Prediction of Strength Properties of Filling Packets in Selected Cooling Towers
Source: Polymers (Basel). 2021 Nov 6;13(21):3840. doi: 10.3390/polym13213840 (PMC8587418; doi:10.3390/polym13213840)
Supplement: Supplementary file 1 [file polymers-13-03840-s001.zip › polymers-1409878-supplementary.pdf]

Supplementary Material

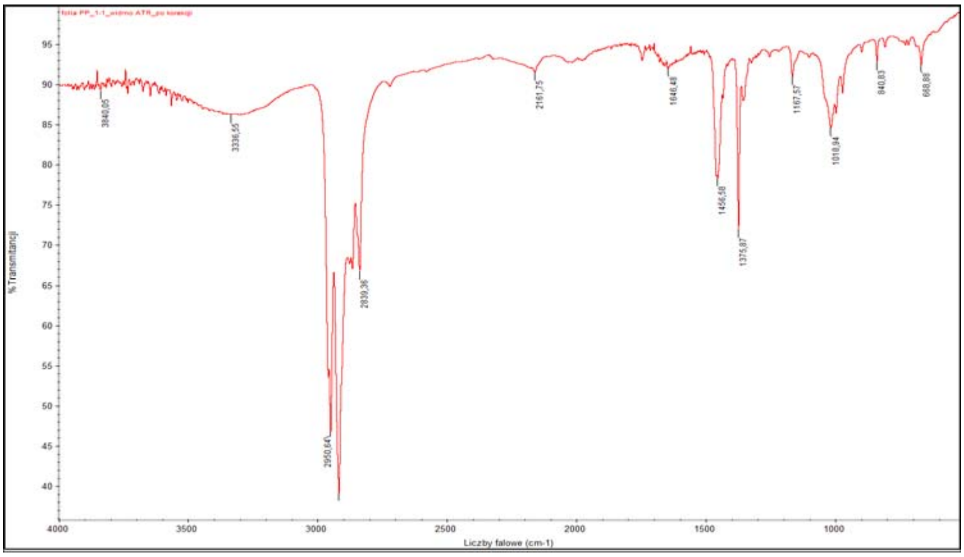

Figure S1. The spectrum of CT1_LS

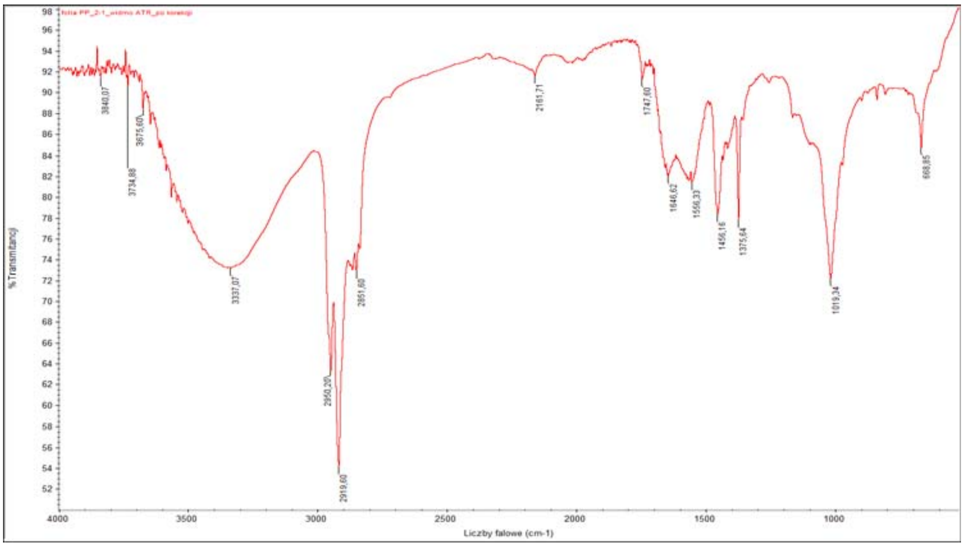

Figure S2. The spectrum of CT1_C

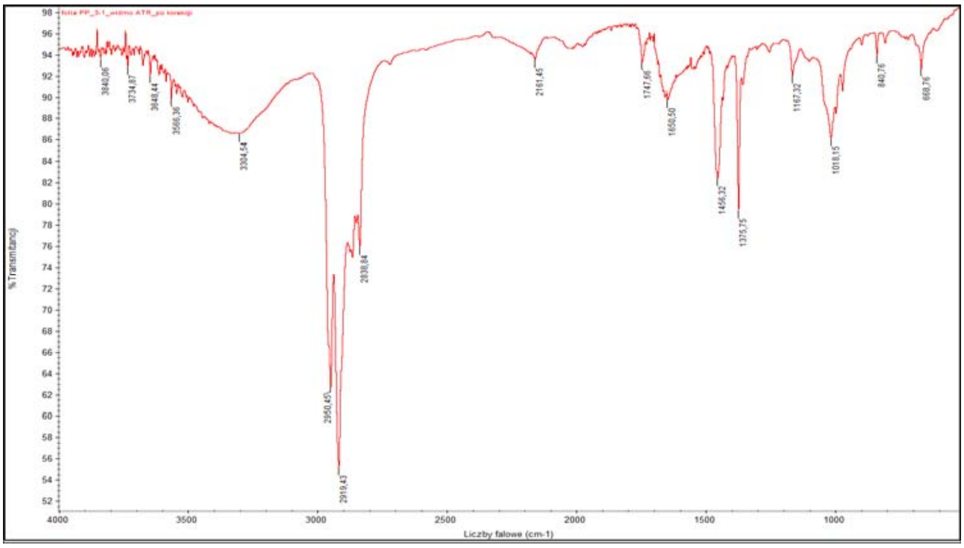

Figure S3. CT1_RS spectrum

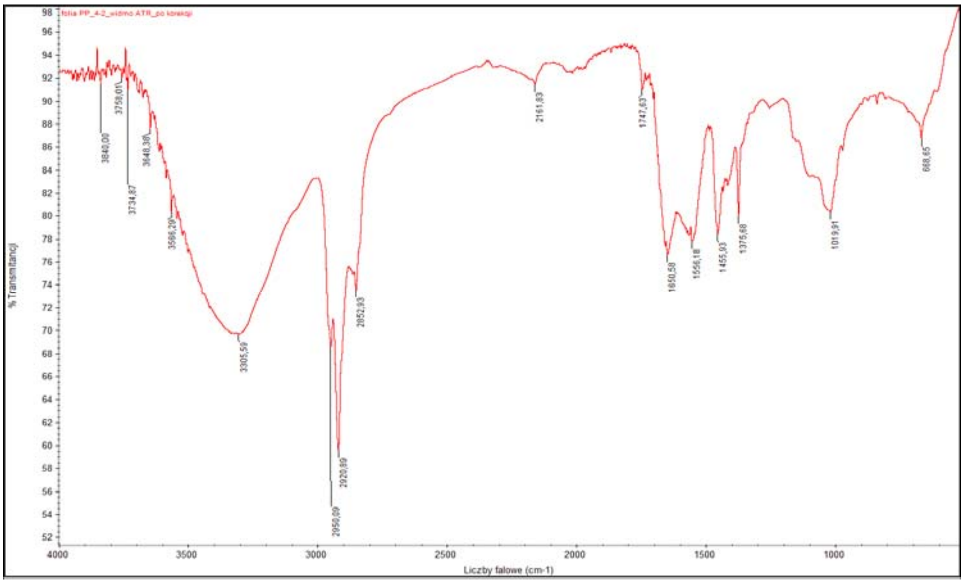

Figure S4. The spectrum of CT2_C2

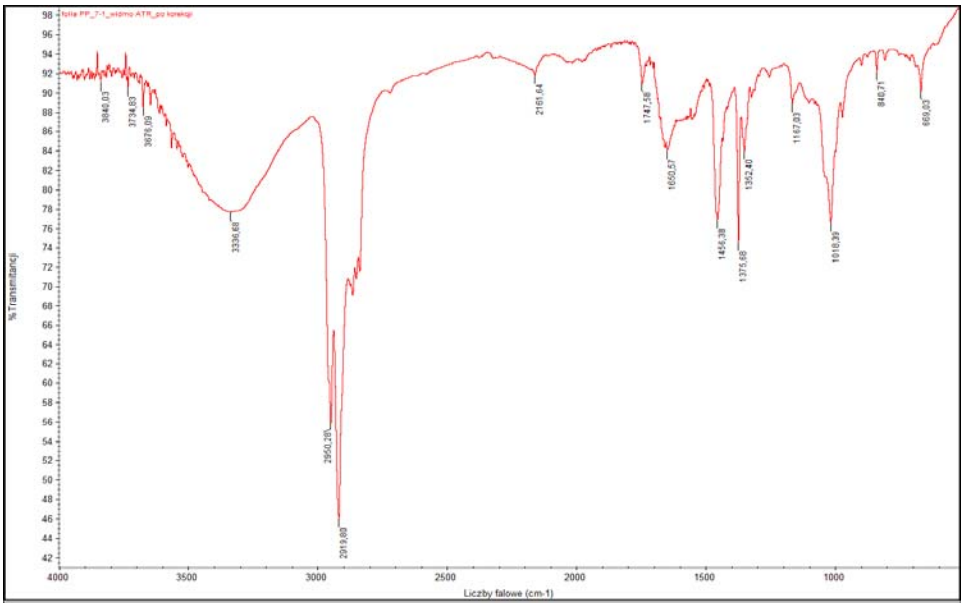

Figure S5. CT2_RS spectrum

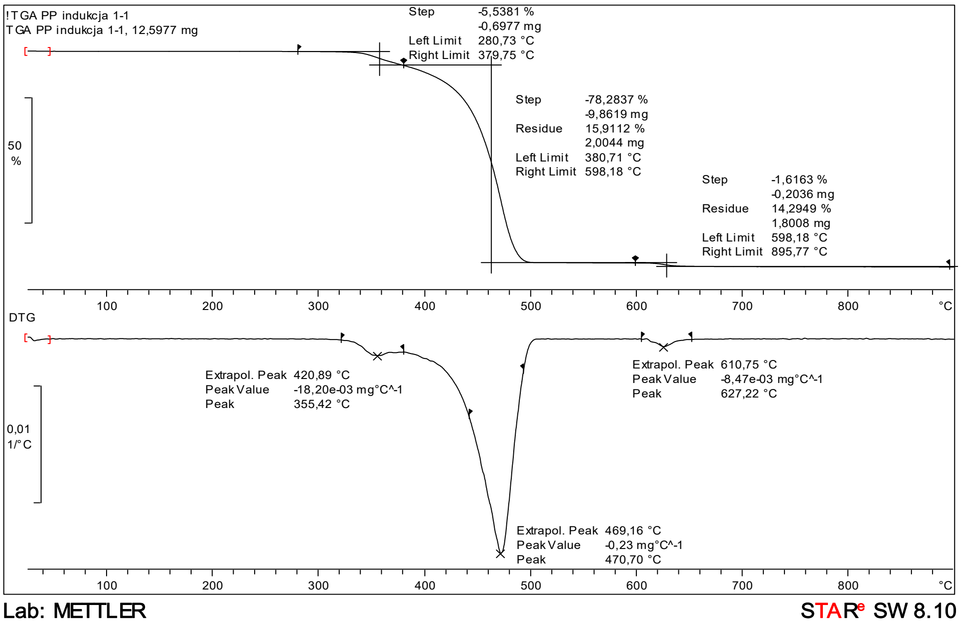

Figure S6. Dependence of sample mass on temperature increasing with constant velocity in the TGA CT1_LS tests

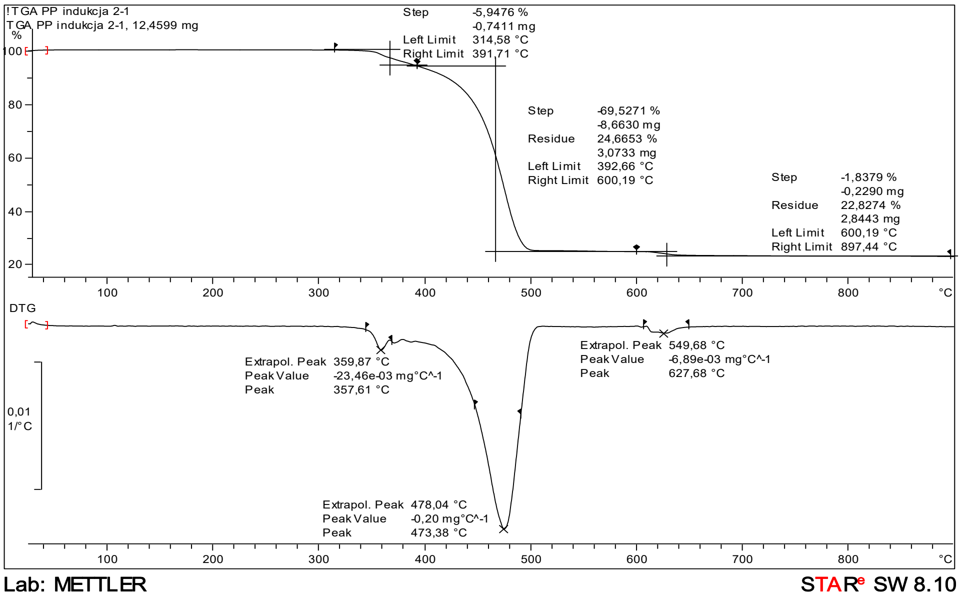

Figure S7. Dependence of sample mass on temperature increasing with constant velocity in the TGA CT1_C tests

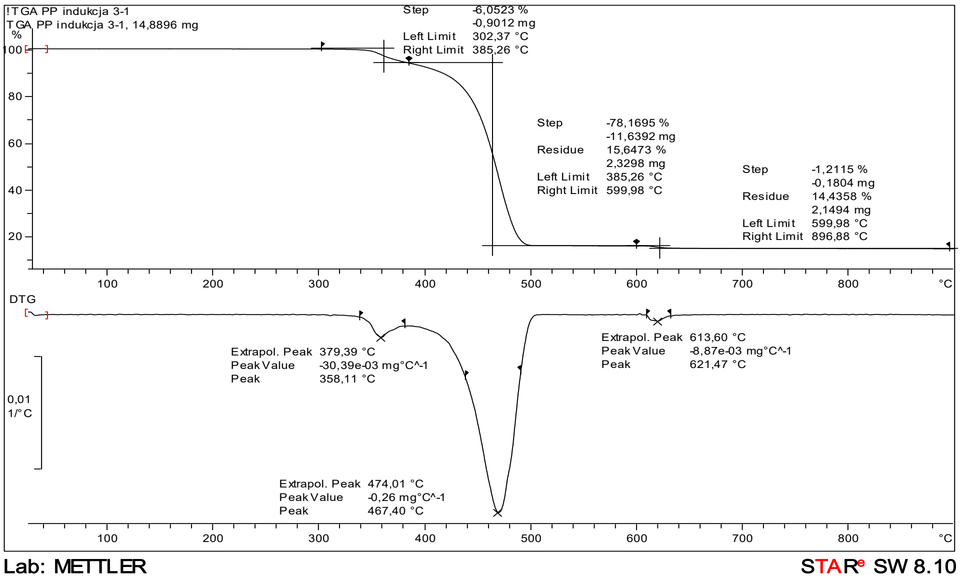

Figure S8. Dependence of the sample mass on the temperature increasing with a constant speed in the TGA CT1_RS tests

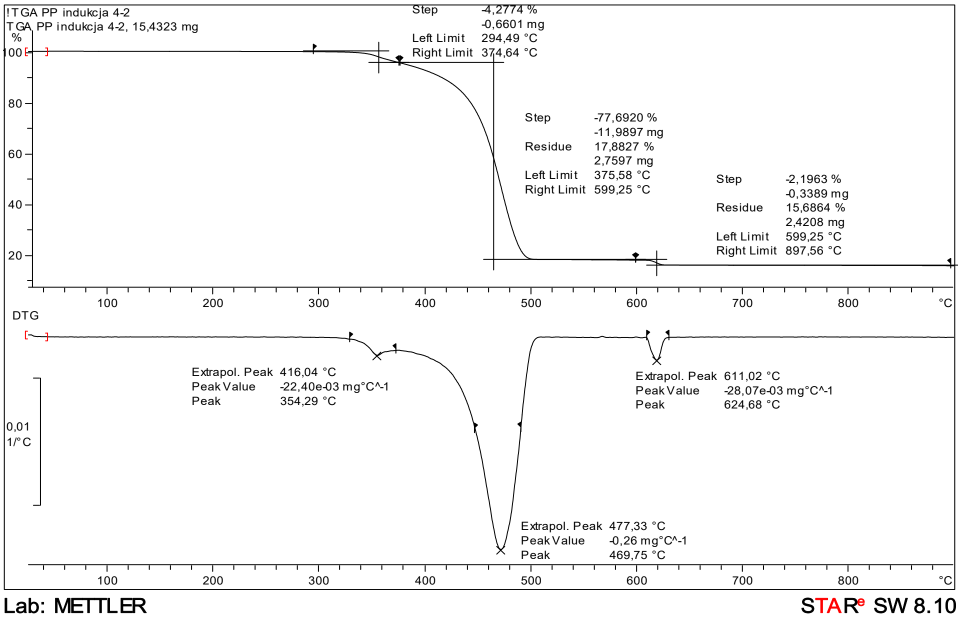

Figure S9. Dependence of sample mass on temperature increasing with constant velocity in TGA CT2_C tests

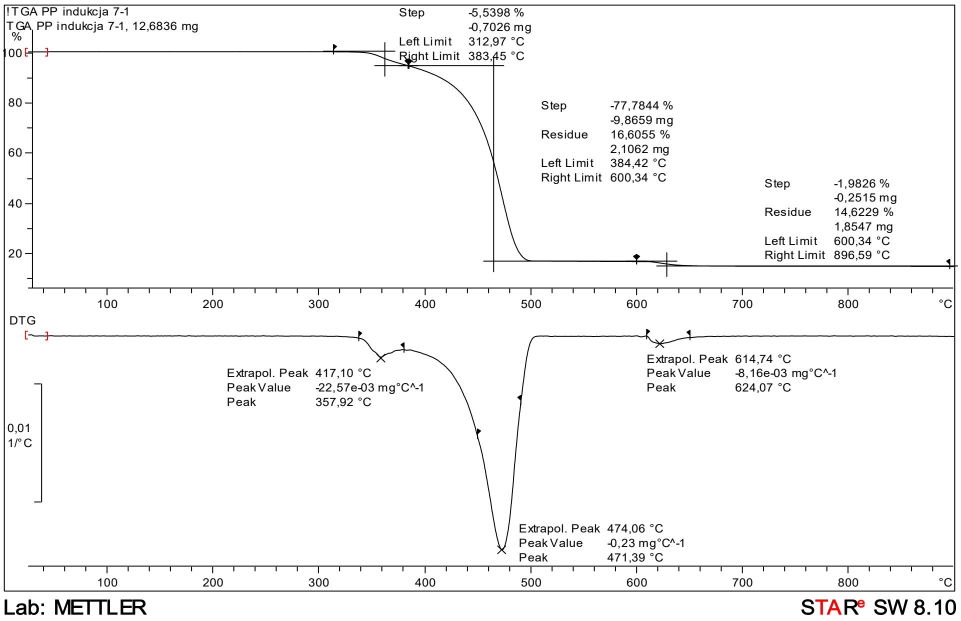

Figure S10. Dependence of sample mass on temperature increasing with constant velocity in TGA CT2_RS tests
